# E-consults: an effective way to decrease clinic wait times in rheumatology

**DOI:** 10.1186/s41927-020-00152-5

**Published:** 2020-10-15

**Authors:** Veena Patel, Diana Stewart, Molly J. Horstman

**Affiliations:** 1 Division of Rheumatology, Department of Medicine, University of Texas at Austin, Dell Medical School, 1601 Trinity St., Bldg B, Stop Z0900, Austin, TX 78712 USA; 2 Section of General Internal Medicine, Department of Medicine, Baylor College of Medicine, Houston, TX USA; 3 VA HSR&D, Center for Innovations in Quality, Effectiveness and Safety, Michael E. DeBakey VA Medical Center, Houston, TX 77030 USA; 4 Section of Health Services Research, Department of Medicine, Baylor College of Medicine, Houston, TX USA; 5 VA Quality Scholars Coordinating Center, IQuESt, Michael E. DeBakey VA Medical Center, Houston, TX USA

**Keywords:** Quality improvement, Telemedicine, Referral and consultation, Rheumatology

## Abstract

**Background:**

To evaluate the effect of E-consults on wait times and resource utilization for positive antinuclear antibody (ANA) referrals in outpatient rheumatology.

**Methods:**

We conducted a pre-post study of E-consult implementation for positive ANA referrals. We retrospectively reviewed “positive ANA” referrals from 1/2015–3/2017. A statistical process control chart was created to display monthly average wait times for in-person clinic visits and to identify special cause variation. Final diagnoses, wait times and resource utilization were recorded and compared between E-consults and in-person referrals.

**Results:**

There were 139 referrals for positive ANA with 126 occurring after E-consult implementation in August 2015. Forty-four percent (55/126) of referrals were E-consults; 76% did not have an in-person visit after initial electronic rheumatology recommendation. A control chart demonstrated special cause variation in the form of a shift from June 2016 – January 2017, suggesting a temporal association between decreased wait times and the implementation of E-consults. Eleven patients were diagnosed with ANA-associated rheumatic disease; the majority of patients (73%, 86/139) did not have a rheumatologic diagnosis. Overall E-consults utilized more labs than in-person visits, but this was not statistically significant. In-person visits utilized more imaging studies, which was statistically significant.

**Conclusion:**

E-consults are an effective way to address positive ANA consults without significant increase in resource utilization and were temporally associated with decreased wait times for in-person visits.

## Background

The term *telehealth* refers to multiple modalities to provide care remotely using some form of technology. Due to the COVID-19 pandemic, there has been a rapid increase in telehealth usage in 2020 [1]. One type of telehealth format, the electronic consult (E-consult), has been utilized by healthcare systems in the United States and internationally for many years [2, 3] as a way to help patients gain access to specialty care. E-consults are an asynchronous form of communication between referring provider and specialist through a shared EHR (electronic health record) or web-based platform that entails the specialist reviewing chart data and replying with recommendations electronically [1, 3]. E-consults have facilitated a decrease in face-to-face visits, increased access to care, and improved provider satisfaction [3]. E-consults also affected wait times, from shortening clinic wait times or the perception of wait times, but multiple contributing factors in real-world settings have made it difficult to precisely assess this impact [3, 4].

Prior studies have shown the benefit of synchronous telehealth modalities within rheumatology such as videoconference visits [5–9], but there is little published about the impact of asynchronous E-consults. Scheibe et al. [10], reviewed the impact of another type of asynchronous telehealth format, the pre-consult exchange, which uses a similar strategy as E-consults, but with more integrated communication between the referring provider and specialist. One study evaluated E-consults in rheumatology and showed advantages including quicker turn-around times, a decrease in face-to-face visits, and increased referring provider satisfaction. Common reasons E-consults were utilized in this study included treatment questions, questions about whether a patient required an in-person evaluation, and interpretation of a positive laboratory test [2].

A positive antinuclear antibody (ANA) test is a common reason for referral to rheumatology. The ANA is a non-specific test and is often checked in the absence of clinical signs and symptoms of an ANA-associated rheumatic disease (AARD). Studies have shown that a positive ANA resulting in a diagnosis of an AARD is low (11% or less) [11, 12]. Another study reported when an ANA was checked without using proper clinical criteria, 88% of positive cases had no systemic rheumatic disease diagnosis [13]. ANA positivity is present in the general healthy population [14, 15], and its prevalence has increased in the past 25 years [16]. Given the increase in prevalence in healthy individuals coupled with the shortage of rheumatologists in the US [17] sending referrals for positive ANA is an inefficient use of limited resources.

Finding ways to address positive ANA referrals promptly, such as through the use of E-consults, can improve access for patients requiring in-person rheumatology evaluation. This project aims to evaluate the resource utilization of E-consults and their effect on wait times for positive ANA referrals in a rheumatology clinic.

## Methods

We conducted a pre-post study of E-consult implementation for positive ANA referrals. Our outcomes included wait times, diagnostic tests ordered, and patients’ final diagnoses.

The Michael E. DeBakey VA Medical Center is a tertiary health care center consisting of inpatient and outpatient medical services caring for 113,000 Veterans in Southeast Texas. An EHR is used by all providers throughout this system and any provider within the system can order a referral for specialty care. The referring provider places an order for a referral to rheumatology in the EHR; reason for consult is entered into a free text box within the referral order. Starting in August 2015, the referring provider had the option of selecting either “E-consult” or “in-person visit” when placing a referral to rheumatology.

The referral review process was completed by one faculty rheumatologist who reviewed all referral requests and determined if the type of referral selected by the referring clinician (in-person visit or E-consult) was appropriate. The reviewer had the option to switch the type of consult if needed. For E-consults, the reviewer electronically replied through the EHR to the referring clinician with recommendations after reviewing the patient’s electronic chart. Rheumatology staff scheduled clinic appointments for in-person referrals.

We retrospectively reviewed positive ANA referrals to outpatient rheumatology from January 1, 2015, to March 31, 2017. We defined a positive ANA as a result with a titer greater than or equal to 1:40 (our lab reference range) by indirect immunofluorescence. Through chart review, we collected demographic data, referral information, wait times, labs, and imaging ordered during the first rheumatology evaluation and final diagnoses. Chart review was done using a standard data abstraction form with standard definitions of every type of extracted data and standard locations for identifying this data from the EHR.

We defined wait time for an in-person visit as the time from referral placement to the rheumatology clinic appointment. E-consult wait time was defined as the time from referral placement to the rheumatologist’s initial electronic response. We calculated the average monthly wait time for a positive ANA in-person visit and graphed the results using an XmR statistical process control chart to assess for special cause variation [18]. An unpaired T-test was used to compare wait times and resource utilization between visit types in total, and for each final diagnosis category.

Resource utilization was defined as rheumatologic labs and imaging ordered during the first rheumatology evaluation. The following labs were included in data collection: rheumatoid factor, anti-cyclic citrullinated peptide (anti-CCP), anti-Ro (SS-A), anti-La (SS-B), anti-Smith, anti-nRNP, anti-double stranded DNA (dsDNA), complement C3 and C4, Scl-70, sedimentation rate, C-reactive protein, antiphospholipid antibody panel, antineutrophilic cytoplasmic antibodies, cryoglobulins, aldolase, HLA-B27, uric acid, and anti-Jo1. Imaging studies recorded included joint X-Rays, body or joint computed tomography (CT) scans, and joint magnetic resonance imaging (MRI) scans. Resource utilization was calculated as the mean per person and for each final diagnosis category.

At the time of review, patients’ final diagnoses were recorded from the assessment and plan section of the rheumatologist’s most recent clinic note in the EHR. Diagnoses were organized into the following categories: ANA-associated rheumatic disease (AARD), other rheumatic disease (ORD), no rheumatic disease, or no diagnosis at the time of review. AARD included the following diagnoses: systemic lupus erythematosus (SLE), Sjogren’s syndrome, scleroderma, mixed connective tissue disease (MCTD), polymyositis (PM), dermatomyositis (DM), undifferentiated connective tissue disease (UCTD), and drug-induced lupus [15]. ORD included diseases that would require routine rheumatology follow-up, but in which an ANA is not used for the diagnosis.

This study was reviewed by the Institutional Review Board (IRB) and the local Veterans Affairs Research and Development Committee and designated as a quality improvement project which did not require formal IRB approval.

## Results

**Fig. 1:**
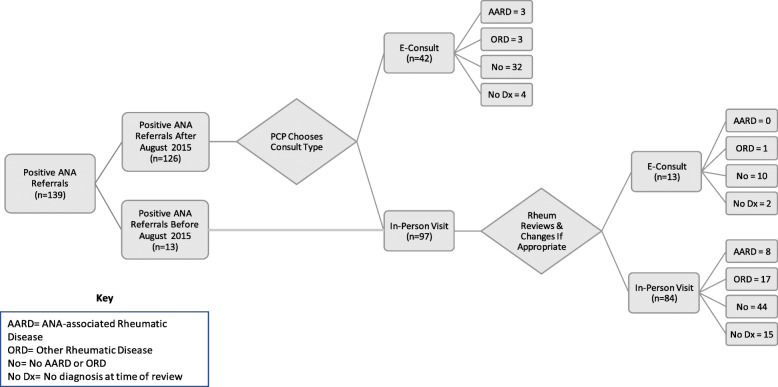
The journey of positive ANA referrals through the referral system and the final diagnosis category. Primary care providers (PCP) choose the initial consult method (E-consult or in-person visit). A rheumatologist reviews all consults before appointments are scheduled and can change clinic visits to E-consults (or vice versa) if their chart review deems this appropriate. No in-person visits were changed to E-consults during our project

There were 139 positive ANA referrals within our system from January 1, 2015, to March 31, 2017. The majority of patients were white (*n* = 78, 56%) and male (*n* = 94, 68%), with an average age of 54 years (Table 1). Of the 139 positive ANA referrals, 126 occurred after E-consult implementation in August 2015. While referring providers selected the initial consult type, 13% of in-person referrals (13/97) were switched to E-consults by the reviewing rheumatologist (Fig. 1). No E-consults were initially switched to in-person visits during the study period.

**Table 1:** Demographic characteristics of patients

Characteristics of Patients Referred for Positive ANA	
	n (%)
**Sex**	
Female	45 (32%)
Male	94 (68%)
**Race**	
White	78 (56%)
Black	51 (37%)
Hispanic	8 (6%)
AI/Hawaiian	1
Asian	1
**Age**	
Age Average	54
Standard Dev	14
Age Range	28–89

E-consults were utilized in 44% (55/126) of patients with an average response time of 1.7 days. An in-person visit was deemed unneeded after the initial rheumatologic recommendation for 76% (42/55) of E-consults. During the duration of this study, none of these E-consults were subsequently scheduled for an in-person visit after initial E-consultation.

**Fig. 2:**
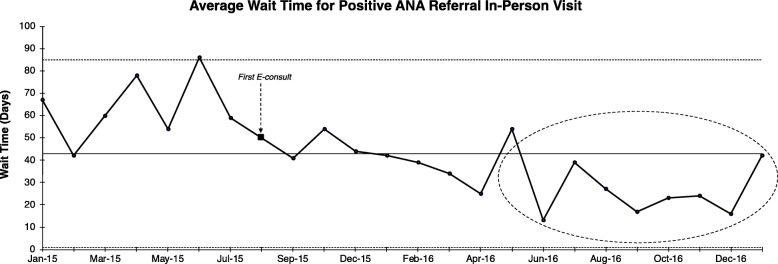
Statistical process control chart displaying average monthly wait time for in-person positive ANA referrals before and after the initiation of E-consults. The circle indicates special cause variation with > 6 points below the central line of tendency, suggesting that the introduction of E-consults may be associated with decreased wait times

The average in-person visit wait time for a positive ANA referral decreased from 64 to 34 days after E-consult implementation (*p* < 0.001). Special cause variation [18] was identified as a shift in the control chart from June 2016 to January 2017 (Fig. 2).

Eleven Veterans (8%) had a diagnosis of AARD: SLE (*n* = 5), limited systemic sclerosis (*n* = 1), UCTD (*n* = 1), MCTD/SLE overlap (*n* = 1), Sjogren’s syndrome (*n* = 1), DM (*n* = 1) and drug-induced lupus (*n* = 1). ORDs were diagnosed in 21 Veterans and included: gout, non-radiographic ankylosing spondylitis, fibromyalgia, sarcoidosis, cryoglobulinemia, polymyalgia rheumatica, and rheumatoid arthritis. The majority of referrals resulted in no rheumatic diagnosis (86/139, 62%). Of the 13 referrals that were switched from in-person visits to E-consults by the reviewing rheumatologist, one was ultimately diagnosed with rheumatoid arthritis and secondary Sjogren’s syndrome. The E-consult for this patient was used to order more specific lab work before the in-person visit was scheduled.

E-consults ordered more labs overall, but this was not statistically significant. In-person visits ordered more imaging overall, which was statistically significant (Table 2). The most common labs ordered by E-consults were anti-Smith (*n* = 32), anti-Ro/La (*n* = 31), anti-nRNP (*n* = 31), and dsDNA (*n* = 29). The most common labs ordered by in-person visits after the implementation of E-consults were anti-Ro/La (*n* = 22), C3/4 (*n* = 22), dsDNA (*n* = 21), anti-Smith (*n* = 19).

**Table 2:** Resource utilization between E-consults and in-person visits for different diagnosis groups. Overall, more lab tests were ordered per person by E-consults, but this was not statistically significant. In-person visits ordered more imaging studies. Labs and imaging studies are reported as a mean per person with the range listed in parentheses

Resource Utilization of Positive ANA Referrals			
	In-person Visits	E-consults	***p***-value
	*n* = 84	*n* = 55	
Labs (per person)	3.2 (0–9)	3.9 (0–10)	0.11
Imaging Studies (per person)	1.1 (0–9)	0.1 (0–2)	0.0003
**Final Diagnoses**			
**AARD**	8	3	
Labs	3.1	5.7	0.11
Imaging Studies	1.8	0	0.37
**ORD**	17	4	
Labs	3.5	1.8	0.23
Imaging Studies	1.8	0	0.15
**No Rheumatic Disease**	44	42	
Labs	3.1	3.9	0.18
Imaging Studies	0.6	0.1	0.01
**No Diagnosis (at time of review)**	15	6	

*AARD* ANA-associated rheumatic disease, *ORD* Other rheumatic disease

## Discussion

E-consults are an effective way to address ANA referrals quickly and improve access to rheumatology for patients requiring in-person evaluation without a significant increase in resource utilization.

The effect of E-consults on wait times in prior studies have been variable from a decrease in wait times [4] to no change [2, 10]. In our study, detecting special cause variation through a control chart suggests a temporal association between E-consult introduction and decreased wait times. We know of no changes that occurred in the rheumatology clinic at that time that would otherwise contribute to the decreased wait time. The reduction in wait time for in-person visits has other potential positive downstream effects including improving value for healthcare systems and reducing patient costs by decreasing unnecessary travel and time off work for in-person appointments.

In our study, E-consults utilized more lab tests than in-person visits, but this was not statistically significant. We reported 7 E-consults which resulted in a rheumatic diagnosis (3 AARD and 4 ORD) during our study. These cases used the E-consult to initiate lab work-up before an in-person visit was scheduled. These patients were subsequently followed up in clinic. There are certain cases, such as these, when an in-person visit is needed after E-consult and efficiencies are gained by initiating work-up through the E-consult, making the in-person visit more productive. Given the non-specific nature of the indirect immunofluorescence ANA test [11, 15], E-consults, for this reason, may also require more specific lab data before warranting an in-person evaluation.

In our study, 76% of positive ANA referrals did not have further in-person follow-up with rheumatology after the initial E-consult. A prior study in rheumatology showed that 38% of E-consults avoided face-to-face visits [2]. Focusing exclusively on one type of consult: the positive ANA, may explain our higher rate of evaluation by E-consults alone. Also, our patient population of predominantly older white men, are not a high-risk group for AARD and likely had an ANA ordered without clinical signs and symptoms of AARD. Anti-DFS70 antibodies are commonly positive in healthy individuals and are not likely indicative of AARD, even at high titers [19–21]. Incorporating anti-DFS70 antibodies into the evaluation of positive ANA E-consults could be a potential way to avoid unnecessary testing [19] and give reassurance that a patient will not likely develop an AARD over time [20]. The average rheumatology response time to E-consult in our study was similar to prior studies using asynchronous telehealth modalities [2, 10].

Telehealth has been identified as a viable option to continue providing timely care during the current COVID-19 pandemic [1] and many providers have quickly adapted to using telemedicine modalities including E-consults. Not only are E-consults a way to continue care during a quarantine period, but as our results indicate, they are also a considerable way to decrease in-person rheumatology wait times.

Our study is limited by its small scope and short duration. We do not have long-term follow-up data to see if resolved E-consults eventually needed an in-person visit or developed an AARD. We also do not have information about the decision-making process or criteria that was used when reviewing an E-consult, future projects could consider evaluating this to improve or standardize the E-consult process. Also, final diagnoses were based on evaluating the rheumatologist’s clinic note and there was no confirmation by a second rheumatologist of the diagnosis. This study may not be generalizable for health care systems that receive referrals from outside sources and those that do not share an EHR or web-based platform with their referring providers. We also focused on a subset of referrals and do not know if wait times for all types of referrals improved.

## Conclusions

With the ongoing COVID-19 pandemic and surge in telehealth practices, E-consults will likely continue to be incorporated into specialty care, including rheumatology. Our study shows that E-consults are an effective way to address positive ANA referrals without a significant increase in resource use, so patients that require in-person evaluation can be seen in a timely manner. Rheumatologists considering continuing or starting telehealth initiatives may consider focusing on positive ANA referrals to improve wait times for in-person visits.

## Data Availability

The datasets generated during and/or analyzed during the current study are not publicly available due to this work being designated as quality improvement but are available from the corresponding author on reasonable request.

## Abbreviations

ANA: Anti-nuclear antibody
E-consult: Electronic consult
EHR: Electronic health record
AARD: ANA-associated rheumatic disease
Anti-CCP: Anti-cyclic citrullinated peptide
SS-A: Anti-Ro
SS-B: Anti-La
dsDNA: Anti-double stranded DNA
C3: Complement C3
C4: Complement C4
CT: Computed tomography
MRI: Magnetic resonance imaging
ORD: Other rheumatic disease
SLE: Systemic lupus erythematosus
MCTD: Mixed connective tissue disease
PM: Polymyositis
DM: Dermatomyositis
UCTD: Undifferentiated connective tissue disease
IRB: Institutional Review Board
